# Photoperiod-dependent transcriptional modifications in key metabolic pathways in *Coffea arabica*

**DOI:** 10.1093/treephys/tpaa130

**Published:** 2020-10-20

**Authors:** Doâa Djerrab, Benoît Bertrand, Jean-Christophe Breitler, Sophie Léran, Eveline Dechamp, Claudine Campa, Célia Barrachina, Geneviève Conejero, Hervé Etienne, Ronan Sulpice

**Affiliations:** Centre de coopération internationale en recherche agronomique pour le développement (CIRAD), UMR IPME, F-34398 Montpellier, France; UMR IPME, Université de Montpellier, CIRAD, IRD, F-34398 Montpellier, France; Centre de coopération internationale en recherche agronomique pour le développement (CIRAD), UMR IPME, F-34398 Montpellier, France; UMR IPME, Université de Montpellier, CIRAD, IRD, F-34398 Montpellier, France; Centre de coopération internationale en recherche agronomique pour le développement (CIRAD), UMR IPME, F-34398 Montpellier, France; UMR IPME, Université de Montpellier, CIRAD, IRD, F-34398 Montpellier, France; Centre de coopération internationale en recherche agronomique pour le développement (CIRAD), UMR IPME, F-34398 Montpellier, France; UMR IPME, Université de Montpellier, CIRAD, IRD, F-34398 Montpellier, France; Centre de coopération internationale en recherche agronomique pour le développement (CIRAD), UMR IPME, F-34398 Montpellier, France; UMR IPME, Université de Montpellier, CIRAD, IRD, F-34398 Montpellier, France; UMR IPME, Université de Montpellier, CIRAD, IRD, F-34398 Montpellier, France; IRD, UMR IPME, F-34394 Montpellier, France; MGX, Biocampus Montpellier, CNRS, INSERM, University of Montpellier, 34000 Montpellier, France; BPMP, University of Montpellier, CNRS, INRAE, Montpellier SupAgro, Montpellier, France; Centre de coopération internationale en recherche agronomique pour le développement (CIRAD), UMR IPME, F-34398 Montpellier, France; UMR IPME, Université de Montpellier, CIRAD, IRD, F-34398 Montpellier, France; National University of Ireland, Plant Systems Biology Lab, Ryan Institute, School of Natural Sciences, University Road, Galway H91 TK33, Ireland

**Keywords:** Arabidopsis, coffee, core clock, photoperiod, rhythmic genes, transcriptome

## Abstract

Photoperiod length induces in temperate plants major changes in growth rates, morphology and metabolism with, for example, modifications in the partitioning of photosynthates to avoid starvation at the end of long nights. However, this has never been studied for a tropical perennial species adapted to grow in a natural photoperiod close to 12 h/12 h all year long.

We grew *Coffea arabica L.*, an understorey perennial evergreen tropical species in its natural 12 h/12 h and in a short 8 h/16 h photoperiod, and we investigated its responses at the physiological, metabolic and transcriptomic levels. The expression pattern of rhythmic genes, including core clock genes, was affected by changes in photoperiod. Overall, we identified 2859 rhythmic genes, of which 89% were also rhythmic in *Arabidopsis thaliana L*. Under short-days, plant growth was reduced, and leaves were thinner with lower chlorophyll content. In addition, secondary metabolism was also affected with chlorogenic acid and epicatechin levels decreasing, and in agreement, the genes involved in lignin synthesis were overexpressed and those involved in the flavanol pathway were underexpressed.

Our results show that the 8 h/16 h photoperiod induces drastic changes in morphology, metabolites and gene expression, and the responses for gene expression are similar to those observed in the temperate annual *A. thaliana* species.

Short photoperiod induces drastic changes in gene expression, metabolites and leaf structure, some of these responses being similar to those observed in *A. thaliana*.

## Introduction

The circadian clock provides plants with the ability to anticipate and adapt to daily and seasonal environmental changes. In Arabidopsis (*Arabidopsis thaliana L.*), the main plant model used to study plant biology, day length influences plant fitness, plant growth and development processes through the circadian clock (Roach and Wulff 1987, Green et al. 2002). Plant fitness is partly conferred by circadian rhythms, with changes in circadian rhythms allowing to adapt seasonal changes in day length and allowing survival and fecundity (Green et al. 2002, Rubin et al. 2017).

Variations in day length induce variations in daily amounts of photosynthates available and then influence the regulation of major developmental and physiological processes (Ohara and Satake 2017). In Arabidopsis and other higher plants, photosynthesis is regulated by the circadian clock (Gould et al. 2009, Yarkhunova et al. 2018).

Light input signal is transmitted to the circadian clock via direct interaction of photoreceptors, such as phytochromes with clock components (Yeom et al. 2014). Litthauer et al. (2015), showed that a class of blue photoreceptors, phototropins, are able to maintain circadian oscillation of the photosystem II (PSII) operating efficiency. A correct matching of the circadian clock period with that of the external light–dark cycle confers a substantial photosynthetic advantage (Dodd et al. 2005). Many genes involved in the regulation of the primary metabolism such as *CRY1*, *PHOT1* and *PHOT2* are regulated by the circadian output pathway and show a peak in expression at different times of the day. However, the elucidation of the exact role of the circadian clock on primary metabolism and growth is still ongoing. Such studies are difficult for numerous reasons, e.g., the importance of post-transcriptional and post-translational regulations of central clock genes and the notorious impossibility to quantify most core clock proteins. Also, it is difficult to clearly identify outputs protein targets of clock proteins, due to their usually low turnover which erases diurnal variations, despite their gene expression exhibiting clear diurnal variations. A recent study introduces the concept of ‘translational coincidence’ to explain why genes show diurnal patterns and not their cognate proteins (Seaton et al. 2018). Moreover, in Arabidopsis, the circadian oscillator has been associated with the control of light-harvesting and CO_2_ fixation (Dodd et al. 2014). The production of sugars by photosynthesis is a key metabolic output of the circadian clock as well as likely an input for setting a ‘metabolic dawn’ (Haydon et al. 2013). The circadian clock also influences the production of photoprotective pigments early in the day. Harmer et al. (2000) reported that >20 genes encoding enzymes in the phenylpropanoid biosynthetic pathway were coordinately regulated to peak before dawn. How the circadian clock orchestrates the primary photosynthetic metabolism and the secondary metabolism remains largely unknown. However, the extent to which photoperiod influences daily global transcription has been widely described in Arabidopsis in a large range of photoperiods (Flis et al. 2016). In this species, the phasing of several important clock components is photoperiod-dependent. Taken together, these results obtained on the model Arabidopsis seem to be transferable to temperate species (*Populus*, *Eucalyptus*, *Carica papaya*) or to annual tropical plants (tomatoes, rice, potatoes) (Ibáñez et al. 2010, Müller et al. 2016, Solomon et al. 2010, Zdepski et al. 2008). However, virtually nothing is known regarding the capacity of perennial tropical plants to adapt to changes in photoperiods. These plants naturally grow in photoperiods close to 12 h/12 h and consequently do not face almost any seasonal changes in photoperiods.

Climate change represents a major threat not only for tropical tree crops such as coffee, cocoa, mangos and mangosteen, but also for tropical timber trees (Bunn et al. 2015, Normand et al. 2015, Ounlert and Sdoodee 2015, Oyekale et al. 2009). This is mostly due to the rise of temperature in traditional cultivation areas. One of the possible solutions would be to cultivate such crops at higher latitudes. However, this raises the question of the adaption of tropical perennial plants to short-days during the winter season.

In the present study, we used *Coffea arabica L.*, which is a woody tropical perennial evergreen dicotyledonous species. *Coffea arabica* has its primary center of diversity in the southwestern Ethiopian highlands in an area between 6.50°–14.42°N and 36.12°–44.25°E (Takahashi and Todo 2017). It is an understorey tree growing in the Ethiopian highland forests. In culture, *C. arabica* is grown in many tropical countries both under full sun and under shade in agroforestry systems (Boreux et al. 2016).

In this study, we grew coffee plants under two photoperiod regimes, short-days (8 h/16 h day/night) and the photoperiod coffee encounters in its natural environment, 12 h/12 h. The plants were phenotyped for growth and leaf metabolic content was determined for sugars and some secondary metabolites part of the lignin and flavonoid pathways. The leaf transcriptome was assessed by RNA sequencing (RNAseq) over a diurnal cycle. The aim was to investigate how changes in photoperiods affect the transcriptome globally and to look at specific biological functions important for adaptation to photoperiodic changes, such as photosynthesis and the circadian clock. Finally, we compared transcriptome modifications in *C. arabica*, a tropical plant, with the model plant Arabidopsis.

## Material and methods

### Plant material and cultivation in phytotron chambers

*Coffea arabica* species was represented by cv red Bourbon. Fresh mature seeds were provided by the Nicafrance Experimental Station at La Cumplida (Matagalpa, Nicaragua). After germination, the coffee seedlings were grown in a glasshouse under natural daylight, at a constant temperature of 27 °C for 70 days. Before transferring the plants to phytotrons (43.6458893°N, 3.8684079°E), 30 plants were harvested, and dry weight (103 °C for 24 h) was determined.

Two light:dark (LD) periods were compared in two phytotrons (CRYONEXT, France, model RTH 1200 L). In both phytotrons, temperature, humidity and luminosity were set, respectively, at 22/18 °C day/night, 80% and 600 μmol m^−2^ s^−1^. The *C. arabica* plants were exposed to the intertropical optimal photoperiod of 12 h/12 h (day/night) photoperiod or a short-day photoperiod of 8 h/16 h. For each photoperiod, 30 plants were grown. After 12 weeks of treatment, the dry weights, plant size and number of leaves were measured on 10 plants.

Metabolic and gene expression analyses were performed on the leaves of the terminal young pair. Sampling was performed every 3 h, starting from Zeitgeber time (ZT) 0 (dawn). ZT0 and ZT24 samples were collected in darkness, within 5–10 min before the light switched on. Three replicates were collected for each time point/photoperiod, one replicates corresponding to the leaves of the terminal young pair of one tree. After harvest, the leaves were immediately flash-frozen in nitrogen and stored at −80 °C until RNA extraction or metabolic analyses.

### Chemical analyses

#### Sugars

Sugars (sucrose, fructose, glucose, stachyose, raffinose and maltose) were extracted from 40 mg freeze-dried powder in 3 ml of ethyl alcohol 80% (V/V) containing 0.5 mg ml^−1^ of lactose as an internal standard. After heating (20 min, 80 °C) and centrifugation (4000 r.p.m., 4 °C, 15 min), the supernatant was freeze-dried, dissolved in 9 ml of distilled water and filtered before analysis. Posteriorly, sugars were quantified by High-Performance Anion Exchange Chromatography coupled with Pulsed Amperometric Detection (Dionex Chromatography Co., Sunnyvale, CA, USA) using a CarboPac PA-1 (Dionex) HPLC column, as described by Dussert et al. (2006).

#### Phenolic compounds

After lyophilization, the plant material was ground in a ball mill (TissueLyser II, Qiagen). Extraction was performed by stirring 25 mg of dry powder in 6 ml of MeOH/H2O (80:20, V/V) for 3 h at 4 °C (225 r.p.m., Rotamax 120, Heidolph). After centrifugation (8 min, 3500 r.p.m.), the methanol extract was filtered (Millipore, 0.2 μm porosity) before analysis. Ten milliliters of the extract was quantified using an HPLC system (Shimadzu LC 20, Japan) equipped with a photodiode array detector and composed of an Eclipse XDB C18 (3.5 μm) column (100 mm, 4.6 mm, Agilent), as described in Campa et al. (2017).

#### Chlorophyll extraction

Leaves were ground in liquid nitrogen in a ball mill (Tissue Lyser, Qiagen) and extraction was carried out by stirring 60 mg of leaves in 1.5 ml of acetone 80% (V/V) for 4 h at 4 °C in darkness. After centrifugation at 100,000*g* and 4 °C for 10 min, the supernatant was diluted 10 times in acetone 80% (V/V). The chlorophyll content was calculated using an absorbance spectrophotometer at 645 and 663 nm wavelengths and 80% acetone as control. Chlorophyll content is expressed in milligrams of chlorophyll per gram of fresh leaves and its calculation formula is: Chlorophyll (mg g^−1^) = 8.02 × A663 + 20.20 × A645 (Moran 1982). All quantifications were performed in triplicate.

### Histological observations

Sections of *C. arabica* leaves (terminal young pair) from plants under optimal (12 h/12 h) or short (8 h/16 h) photoperiod conditions were collected and incubated in a fixative solution (1% glutaraldehyde, 2% paraformaldehyde and 1% caffeine in 0.1 M phosphate buffer pH 7.0) for 24 h at 4 °C. Samples were then dehydrated with ethanol and embedded in Technovit 7100 resin (Kulzer Werheim, Germany). The cross-sections (3.5 μm), obtained with a Leica microtome, were oxidized in 1% periodic acid (5 min), washed with distilled water and then stained in the dark for 10 min with Schiff reactant. After washing, sections were stained with Naphtol Blue Black [(CI 20470) 1 g in 7% (v/v)] for 5 min at 60 °C, treated with 7% acetic acid and finally dried at 60 °C for 15 min. The pictures were obtained with a DM6000 Leica microscope (Leica Microsystems, Wetzlar, Germany) or Nikon-Eclipse microscope (Japan) with an objective ×100.

### RNA extraction

For each sample, total RNA extraction from 1 g of leaves was conducted with an improved method from Morcillo et al. (2006) based on Corre et al. (1996). Total RNA was estimated by measuring the optical density of 1 μl extract at 260 nm using a spectrophotometer adapted to a small volume (Nanodrop, ND, Labtech, France).

### RNAseq and bioinformatics

RNAseq was performed by the MGX platform (Montpellier GenomiX, www.mgx.cnrs.fr/), as previously described by Bertrand et al. (2015). The RNAseq library was constructed using the TruSeq RNA sample preparation kit (low throughput protocol) from Illumina. A total RNA aliquot of 1 μg was used to construct the library. The first step in the workflow involves the purification of polyadenylate, containing mRNA molecules using oligo(dT) attached to magnetic beads. After purification, divalent cations are used to fragment mRNA into small fragments at high temperature. SuperScript II reverse transcriptase and random hexamer primers were used to copy the cleaved RNA fragments into the first-strand cDNA. Subsequently, second-strand cDNA synthesis was performed. These cDNA fragments then undergo an end repair process, a single adenine base addition and subsequent adaptator ligation. The product was then purified and enriched with 15 PCR cycles. DNA 1000 Labchip on Bioanalyzer (Agilent) was used to check the final cDNA library and KAPA qPCR kit was used for quantification. For each sequencing lane, four or six libraries were pooled in equal proportions, denatured with NaOH and diluted to 6.5 pM. Following the manufacturer’s instructions, 100 nucleotides single read sequencing was done after the clustering of reads. Image analyses and base calling were performed using the HiSeq Control Software and Real-Time Analysis component (Illumina). The quality of the data was evaluated using FastQC software from the Babraham Institute (http://www.bioinformatics.babraham.ac.uk/projects/fastqc/) and the Illumina software Sequence Analysis Viewer. Using the Illumina algorithm 85 CASAVA (1.8.2 version), 72 bp reads were generated from libraries derived from RNA samples. No *Coffea arabica* reference genome with a high quality of assembly is available. Alignment of the 72 bp reads was conducted using Burrows–Wheeler Aligner (Li and Durbin 2009) and against the *Coffea canephora Pierre ex Froehn* reference transcriptome composed of 25,574 protein-coding gene models (Denoeud et al. 2014). A maximum of two mismatched nucleotides (including gaps) was allowed between the reference and the read. Unmapped and multi-position matched reads were excluded from analyses and SAMtools was used to count the uniquely mapped reads for each gene model. By these criteria, 66–69% of reads mapped uniquely to a genomic location.

### Differential expression analysis between photoperiods and between time points and single enrichment analysis of DEGs

For each sample, we calculated RPKM (reads per kilo base of transcript per million mapped reads) for every transcript (Table S1 available as Supplementary Data at *Tree Physiology* Online). To compare the expression levels, the raw data were normalized with Bioconductor’s DESeq2 v1.14.1 package (Love et al. 2014; Table S2 available as Supplementary Data at *Tree Physiology* Online). Significant differences in expression were identified by DESeq, and significant changes were identified by calculating an adjusted *P*-value (false discovery rate (FDR) <0.05; Benjamini–Hochberg). For each gene, we calculated the fold-change between the same time points of both photoperiods (Table S2 available as Supplementary Data at *Tree Physiology* Online). We also calculated the fold-change between all-time points of 12 h/12 h photoperiod and all-time points of 8 h/16 h photoperiod.

Gene ontology (GO) analysis with the differentially expressed genes (DEGs) was performed using the singular enrichment analysis tool in agriGO v2.0 (Tian et al. 2017) according to the *C. canephora* annotation (Denoeud et al. 2014). Significant GO terms were found using the default FDR *P* ≤ 0.05 cutoff value. Graphs were performed using Excel or R. Heatmap and hierarchical cluster analysis were performed in R 3.2.4 software using the default functions and the gplots package.

### Determination of rhythmic genes

The rhythmic parameters of gene expression, period and phase were determined using JTK_CYCLE algorithm implemented in MetaCycle v1.1.0 (Wu et al. 2016). In order to identify the transcripts showing diurnal oscillations, we analyzed the DESeq2 normalized data with JTK_CYCLE, which uses a non-parametric test (Hughes et al. 2010). JTK_CYCLE was run using a dataset containing 8 h/16 h and 12 h/12 h photoperiods data. We regard transcripts with Benjamini–Hochberg Q values (BH.Q) <0.05 as rhythmic transcripts. JTK-CYCLE was run with a range of periods between 21 and 27 h. We considered as rhythmic genes that were identified by JTK_CYCLE and showed at least one significant difference of expression during the diurnal cycle under the 12 h/12 h and the 8 h/16 h photoperiods (Table S3 available as Supplementary Data at *Tree Physiology* Online).

Genes potentially regulated by evolutionary conserved circadian *cis*-elements (Gbox, EE, CBS, PBX, TBX, SBX, ME and LBS; Table S4 available as Supplementary Data at *Tree Physiology* Online) were identified by an exact string pattern matching approach on the complete promoterome of *C. canephora* genome. This analysis was done considering the 1000 bp upstream sequences of each gene as promoters.

To establish how the photoperiod influences the circadian clock and the metabolism of coffee plants, we focused on genes of the clock and of primary and secondary metabolism for which the sequences were verified against the *C. arabica* genome. Those were identified using BLASTP search against the *C. arabica* phytozome (Goodstein et al. 2012; https://phytozome.jgi.doe.gov/pz/portal.html) database with e value cutoffs of 1e^−15^.

### Validation of RNAseq by qRT-PCR analysis

Validation of RNAseq studies is frequently performed by qRT-PCR. Based on the literature, we targeted three of the circadian clock key genes from *C. canephora CcLHY* (Cc02_g39990), *CcGI* (Cc10_g15270) and *CcLUX-ARRYTHMO* (Cc06_g20160), two genes involved in chlorophyll biosynthesis *CcPOR1A* (Cc05_g12370) and *CcPOR1B* (Cc05_g06850) and two genes involved in starch degradation *CcISA3* (Cc10_g06640) and *CcGWD1* (Cc11_g15490). cDNA synthesis and PCR experiments were carried out, as previously described by Marraccini et al. (2012). All reactions were performed in triplicate for each of the three biological replicates in qRT-PCR. Primers were designed using Primer3Plus online software (http://www.bioinformatics.nl/cgi-bin/primer3plus/primer3plus.cgi). By analyzing the *T_m_* (dissociation) of amplified products, the specificity of the PCR products generated for each set of primers was verified. PCR efficiency (*E*) was estimated using absolute fluorescence data captured during the exponential phase of amplification of each reaction with the equation (1 + *E*) = 10^(−1/slope)^ (Ramakers et al. 2003). Expression levels were calculated by applying the formula (1 + *E*)^−ΔΔ*C*t^, where Δ*C*t, target=*C*t, targetgene−*C*t, *CaGAPDH* and ΔΔ*C*t =Δ*C*t, target−Δ*C*t, references sample, with the *T*_0_ sample being used as references for each construct. The expression level of the *CaGAPDH* gene (GB accession number GW445811) was used as an endogenous control to normalize the expression level. The relative log expression normalized data estimated from RNAseq had a high correlation with that from the qRT-PCR (*r* = 0.79).

### Regulatory networks inference

In order to identify the interactions between five core clock genes (*CaGI*, *CaLHY*, *CaELF4*, *CaPRR5* and *CaPRR7*) and genes involved in four metabolic pathways, networks inference was performed using the R package TDCor version 0.1–2 (Lavenus et al. 2015). The parameters used to run TDCor are described in Table S5A available as Supplementary Data at *Tree Physiology* Online. We performed four analyses corresponding to four pathways grouped in four datasets (namely, chlorophyll biosynthesis and degradation, light phase of photosynthesis, the second phase of photosynthesis, starch synthesis and degradation), and we established inference with each core clock genes. Datasets contain only genes showing diurnal oscillations and at least one significant difference in expression (*P*_adj_ < 0.05) between both photoperiods during the diurnal time course. We inferred networks from each of the two photoperiods. The networks considered were those that (i) showed at least one existing interaction (bootstrap >79.4%) between the circadian gene under study and at least one gene of the metabolic pathway under consideration, and (ii) this interaction was present under both the 12 h/12 h photoperiod and 8 h/16 h photoperiod (Table S5B available as Supplementary Data at *Tree Physiology* Online). Then the regulatory networks were visualized using Cytoscape v3.6.1. (Cline et al. 2007; Table S5C available as Supplementary Data at *Tree Physiology* Online).

## Results

### Photoperiod induces changes in plant growth, leaf structure and chlorophyll content

**Figure 1.**
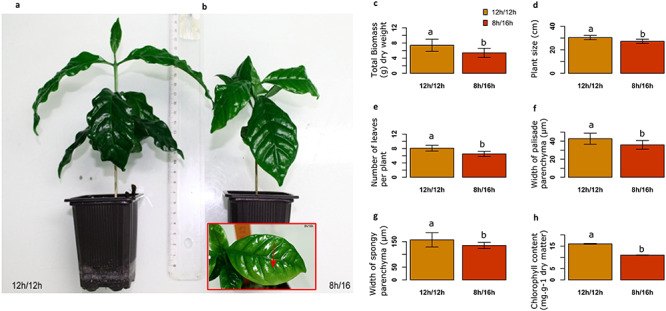
Short-days limit growth affects leaf morphology and chlorophyll content. *Coffea arabica* plants were grown for 12 weeks in 12 h/12 h and 8 h/16 h day/night photoperiods under 600 μmol m^−2^ s^−1^ light intensity and 22/18 °C day/night; we noted a slight photobleaching on young leaves of coffee cultivated under short-day (b) in contrast with plants cultivated under 12 h/12 h photoperiod (a). Influence of photoperiod on biomass (c) and plant size (d), number of leaves/plant (e), width of palisade parenchyma (f), spongy parenchyma (g) and chlorophyll content (h) of leaves were measured. In the barplot, the same letter indicates means are not significantly different at *P* ≤ 0.05 (LSD test). Three experimental replicates and nine biological replicates for each of the two photoperiods were considered.

The effect of the photoperiods on plant growth, leaf characteristics and chlorophyll content were examined after 12 weeks of growth in each photoperiod. The plant biomass, size and number of leaves were significantly lower for the plants grown under 8 h/16 h photoperiod than for plants grown under the 12 h/12 h photoperiod (Figure 1). Interestingly, the morphology of the leaves was also affected with a decrease in the width of palisade and spongy parenchyma under short photoperiod, and the chlorophyll content also significantly declined (16 ± 0.2 and 11 ± 0.1 mg g^−1^ dry matter for 12 h/12 h and 8 h/16 h photoperiods, respectively; Figure 1).

### Photoperiod causes more alteration of the transcriptome at the beginning of the day than at the end of day

**Figure 2.**
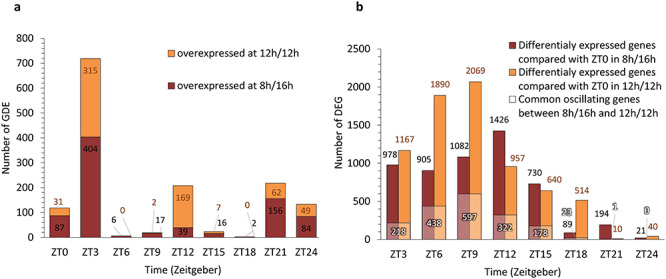
Photoperiod-dependent changes in global gene expression: (a) in response to photoperiod 12 h/12 h, the transcriptional regulation of many coffee leaf genes are down- or up-regulated when compared at the photoperiod 8 h/16 h at different ZT (ZT0 = dawn, ZT12 = dusk) (color brown = down; color orange = up). (b) Comparison of gene expression between ZT0 and all other ZT was made for each of the two photoperiods. Number of DEGs compared with ZT0 at 12 h/12 h photoperiod (orange color) and DEGs compared with ZT0 at 8 h/16 h photoperiod (brown color) among time points is represented. Number of common oscillating genes between 8 h/16 h and 12 h/12 h compared with ZT0 is represented in white color.

RNAseq diurnal time courses were performed on terminal pair leaves of coffee plants grown under the two photoperiods. Changes in photoperiod led to 1446 changes of transcript abundance for 1317 genes during the diurnal cycle (*P*_adj_ < 0.05; Figure 2a; Table S2 available as Supplementary Data at *Tree Physiology* Online). Of these 1317 DEGs, 92 genes showed a fold-change > 2. Main differences in expression between both photoperiods were observed at ZT3 with 700 genes differentially expressed, whereas at ZT6 or ZT18 there were only six and two DEGs, respectively. There were also significant differences at the end of the night and at dawn (ZT21, ZT24 and ZT0) with 208, 133, 118 DEGs, respectively. The number of transcripts that showed photoperiod-dependent changes was comparable at ZT0 and ZT24, confirming the quality of our data.

Comparison of gene expression between ZT0 and all other time points within each photoperiod revealed more DEGs for the 12/12 h photoperiod than for the 8 h/16 h photoperiod. Differentially expressed genes for both photoperiods were mostly observed at the beginning of the day (Figure 2b).

**Figure 3.**
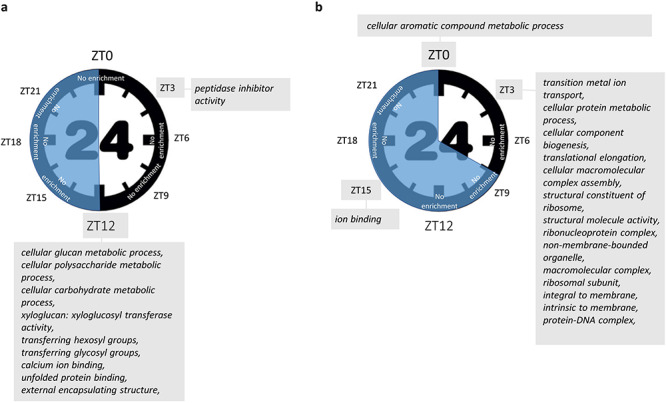
Time-dependent enrichment in functions for both photoperiods. (a) Enrichment GO terms for all DEGs at 12 h/12 h, (b) enrichment GO terms for all DEGs at 8 h/16 h. FDR < 0.05. Photoperiod overexpressed many biological processes as outputs such as cellular aromatic compounds at ZT0 for 8 h/16 h photoperiod or peptidase inhibitor activity for 12 h/12 h photoperiod.

We then performed a GO analysis with all DEGs and plotted the information for both photoperiods (Figure 3a and b). Overall, in short-days, genes related to growth functions are expressed early in the day (ZT3). In contrast, these classes of genes were not enriched during the diurnal cycle for the 12 h/12 h photoperiod and functional classes involved in carbohydrate metabolism were enriched at ZT12.

### Expression of photoreceptors and chlorophyll-binding protein complex are altered by the photoperiod

Because changes in photoperiods led to changes in the morphology of coffee leaves, as well as in their chlorophyll content, we specifically looked at differences in the expression of photoreceptors and genes involved in the light reactions of photosynthesis. Phytochromes (PHYA, PHYB, PHYC), sensing variations in red/far red ratio and the blue light receptors, cryptochromes (CRY1 and CRY2) and phototropins (PHOT1 and PHOT2) are present in coffee plants. In *C. arabica* (‘Ca’ for the prefix), *CaPHYA, CaPHYB, CaPHYC, CaCRY1, CaPHOT1* and *CaPHOT2* did not show significant differences of transcript abundance between the two photoperiods, while *CaCRY2* showed a higher abundance of mRNA after the beginning of the day (ZT3) under the 12 h/12 h photoperiod (Figure S1 and Table S2 available as Supplementary Data at *Tree Physiology* Online).

In contrast, we observed an increase in the expression of genes encoding for proteins part of both photosystems, PSII and PSI at ZT21 (before dawn) for the 8 h/16 h photoperiod compared with the 12 h/12 h photoperiod (Figure S1 available as Supplementary Data at *Tree Physiology* Online), with LHCII chlorophyll a/b binding protein 5 (*CaLHCB5*), P700 apoprotein A1 (*CaPsaA*)*,* PSI subunit II (*CaPsaD*)*,* subunit f of cytb/f (*CaPetA),* subunit b6 of cb/f (*CaPetB*)*,* subunit IV of cytb/f (*CaPetD*)*,* P700 apoprotein A1 (*CaPsaA*)*,* P700 apoprotein A2 (*CaPsaB*)*,* subunit IX of PSI (*CaPsaJ*) and three chlorophyll a-b binding protein, *CaLHCB5*, *CaLHCA4* and *CaLHCB4.2*.

Among the 130 genes present in the *C. arabica* chloroplast genome, no DEGs were observed between ZT0 and ZT18, but 37 genes were overexpressed at ZT21 for the photoperiod 8 h/16 h compared with the 12 h/12 h photoperiod (Tables S2 and S6 available as Supplementary Data at *Tree Physiology* Online). These genes are involved in PSI, PSII, PSI cyclic electron transport (*NDH*), and the cytochrome b/f complex.

Thus, the expression of a number of genes, nuclear and particularly chloroplastic, which are involved in the light reactions of photosynthesis, are affected by a change in photoperiod. Among Calvin–Benson cycle genes, the expression of the Rubisco large subunit (rbcl, gene52) was over four times larger for the photoperiod 8 h/16 h compared with 12 h/12 h (log_2_ fold-change = −2.07; Table S2 available as Supplementary Data at *Tree Physiology* Online).

### Alteration of the circadian clock by photoperiod

**Figure 4.**
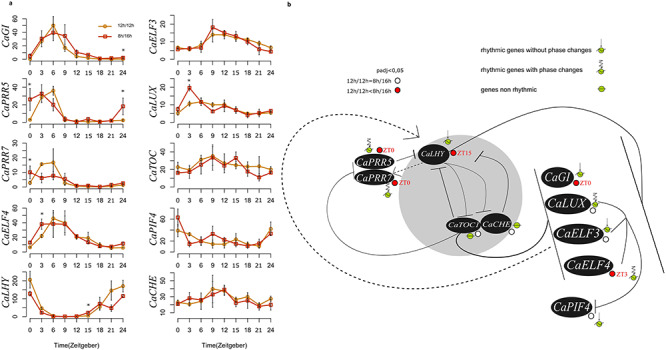
Photoperiod-dependent changes in the expression of core clock genes in 8 h/16 h and 12 h/12 h photoperiods. (a) Photoperiod responses of selected core clock genes. Orange line = 12 h/12 h, red line = 8 h/16 h. ZT0 is equivalent to ZT24. Overall, the patterns of expression observed are identical to those observed in Arabidopsis. Asterisks represent the significant differences of expression occurring between the two photoperiods. *P*-value was adjusted (Benjamini–Hochberg) and significance considered at a threshold of 0.05. Error bars are means ± SEM of three experimental replicates. (b) Expression of the circadian core clock genes and feedback regulations among them. Since we observe expression patterns similar to Arabidopisis ones, we used a figure adapted from Foo et al. (2016) and Singh and Mas (2018), and we made the hypothesis that Arabidopsis genes interactions are globally the same as in the coffee tree. Genes involved are represented in italic and they encode the following proteins: *CaPRR5* = two-component response regulator-LIKE APRR5 (Cc02_g00820); *CaLHY* = protein LHY (Cc02_g39990); *CaELF4* = protein ELF4-LIKE 4 (Cc04_g01390); *CaLUX* = LUX-ARRYTHMO (Cc06_g20160); *CaTOC1* = two-component response regulator-like APRR1 (Cc04_g14990); *CaPIF4* = putative transcription factor PIF4 (Cc05_g00300); *CaPRR7* = two-component response regulator-like APRR7(Cc06_g03460); *CaCHE* = transcription factor TCP7 (Cc07_g19520); *CaELF3* = protein EARLY FLOWERING 3(Cc08_g10110); *CaGI* = protein GIGANTEA (Cc10_g15270). Ca = *C. arabica*. The core clock circadian genes are in the shaded circle. Dashed arrows indicate gene induction and plain lines indicate a gene inhibition. The colored dots near the genes indicate if there is a difference of expression between the two photoperiods (12 h/12 h vs 8 h/16 h); red dots correspond to overexpressed genes at the photoperiod 8 h/16 h and white dots represent genes without difference of expression between the two photoperiods. When the difference of expression occurs between the two photoperiods, the time expressed in hours after the sunrise (ZT) is mentioned next to the dots. Analyses were done with *P*-value adjusted by Benjamini–Hochberg and with a threshold of 0.05.

We observed marked differences in transcript levels between the two photoperiods for five genes of the core clock. At the beginning of the day (ZT0) of *GIGANTEA* (*CaGI*), *CaPRR5* and *CaPRR7* were overexpressed in the plants grown under short-days (Figure 4), while *CaELF4* was overexpressed at ZT3 and *CaLHY* at ZT15. *CaLHY*, *CaGI* and *CaELF3* did not show changes in phase in response to changes in photoperiod, in contrast to *CaLUX*, *CaPRR5* and *CaELF4* (Figure 4). Pairwise phase plots (Figure S2 available as Supplementary Data at *Tree Physiology* Online) between *CaGI* and *CaELF4* or between *CaLHY* and *CaGI* or between *CaELF4* and *CaLHY* kept similar general relationships, whilst marked changes were observed between *CaPRR5* and *CaPRR7* and between *CaELF4* and *CaPRR7*.

### Identification of genes diurnally regulated in coffee and comparison with Arabidopsis

To identify genes with transcripts oscillating during a diurnal cycle, we used the JTK_CYCLE algorithm. JTK_CYCLE provided amplitude, period and phase measurements for the 25,696 genes of the *C. canephora* reference genome (Denoeud et al. 2014). We determined significant oscillations for 4129 genes. This list was then crossed with the list of genes for which we observed at least one significant difference (using Deseq2) at one-time point in each of the two photoperiods, restricting the list to 2859 rhythmic genes showing significant variations in both photoperiods (Table S3 available as Supplementary Data at *Tree Physiology* Online).

**Figure 5.**
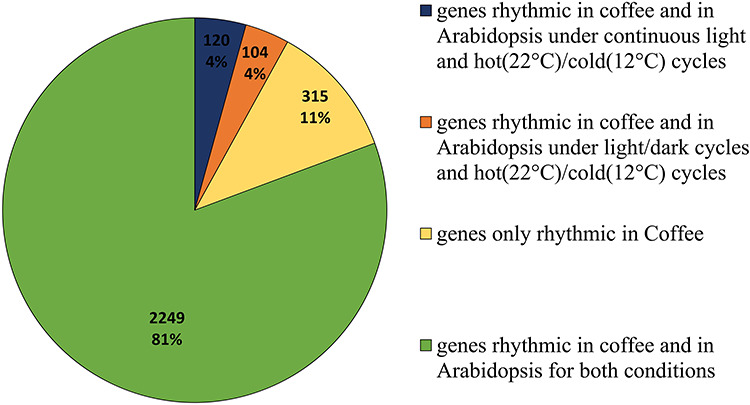
Rhythmic genes are common between coffee and Arabidopsis. We determined a list of rhythmic genes in coffee leaves with JTK-DESeq method. The number of rhythmic genes that have been described as rhythmic or circadian genes in Arabidopsis exposed to hot (22 °C)/cold (12 °C) cycles and continuous light conditions or to both light/dark cycles and to hot(22 °C)/cold (12 °C) conditions was determined. Here are the numbers of rhythmic genes in coffee in each of these conditions or in both of these conditions or the ones not common with these two conditions. Hot (22 °C)/cold (12 °C) cycles corresponds to ‘the average environmental changes across latitude and season for Arabidopsis in its natural habitat’. Arabidopsis circadian genes can be found on DIURNAL website (http://diurnal.mocklerlab.org/). We consider the genes correlated with a threshold of 50%. Lists are provided in Table S3 available as Supplementary Data at *Tree Physiology* Online.

The 2859 coffee rhythmic genes were then blasted against Arabidopsis genome and 2788 orthologues were identified. In order to determine if these genes were also rhythmic in Arabidopsis, we mined lists of Arabidopsis rhythmic genes in datasets available for 11 diurnal and circadian conditions. Data were retrieved from the DIURNAL website (http://diurnal.mocklerlab.org/). For more information, see legend of Figure 5. Altogether, 89% of the coffee rhythmic photo-temperature-dependent genes were also rhythmic in Arabidopsis (Figure 5).

This percentage was similar when using lists of genes showing rhythmicity under light/dark and 22 °C/12 °C cycles or under constant light and 22 °C/12 °C temperature conditions. Consequently, temperature cycles seemed to be responsible for the rhythmicity of the large majority of the genes, as very few additional genes showed rhythmicity when changes in light/dark cycles were associated with variations in temperature.

### Short-day changes the phase of rhythmic transcripts

**Figure 6.**
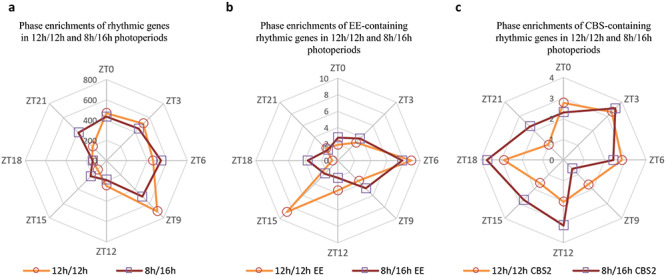
Time-dependent enrichment in core clock elements for both photoperiods: (a) radar chart that represents the number of rhythmic genes with a maximum value of the expression level through photoperiod 12 h/12 h (orange line) and 8 h/16 h (purple line). (b, c) Radar charts that represent the number of rhythmic genes containing *cis*-regulatory modules (EE and CBS) with the maximum value of the expression level through photoperiod 12 h/12 h (orange line) and 8 h/16 h (purple line).

Next, we looked at whether rhythmic genes were affected by photoperiod changes in their phase (Figure 6a; Table S3 available as Supplementary Data at *Tree Physiology* Online). The main changes in the phase of rhythmic genes were observed at ZT9 with more genes peaking under 12 h/12 h than under 8 h/16 h, and at ZT21 where the opposite was observed. Thus, even if our sampling has been done every 3 h and thus we had a lower accuracy compared with the 1 h resolution of Michael et al. (2008), we also observed an anticipation of the gene expression at dawn under short-days. *Cis*-elements motifs that could confer circadian rhythmicity on genes were identified on 52% of the 2859 rhythmic genes, which represents a strong enrichment compared with 27.6% among the 25,696 genes of the coffee genome (*P* < 0.01). The evening cycling genes (Figure 6b) were separated from the morning genes (Figure 6c) by approximately the same period (12 h) as observed by Dodd et al. (2005) and Michael et al. (2008). For genes containing the evening element (EE) *cis*-regulatory motif, we noted changes in phase in response to photoperiods (Figure 6b). We observed a large number of genes peaking at ZT15 under 12 h/12 h while the genes were much more widely distributed under 8 h/16 h, which is consistent with Michael et al. (2008). Other *cis*-elements, such as LUX binding site, also showed striking differences between both photoperiods (Figure S3 available as Supplementary Data at *Tree Physiology* Online). We conclude that photoperiod is a determinant environmental cue for setting the phase of the evening and morning genes in *C. arabica*, similar to Arabidopsis.

### Production of primary and secondary metabolites was altered by photoperiod changes

**Figure 7.**
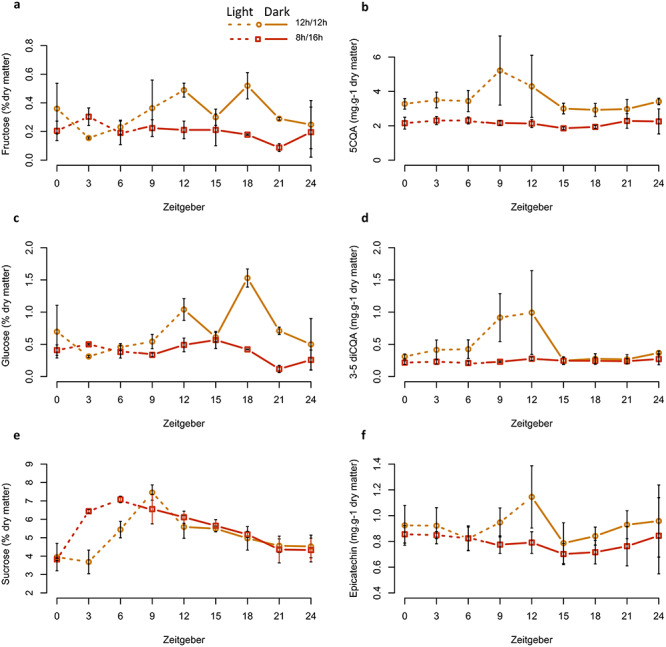
Influence of photoperiod on sugar (sucrose, glucose and fructose) and phenolic (5CQA and epicatechin) contents in leaves. Content in major soluble carbohydrates (a) fructose and phenolics, (b) 5CQA, (c) glucose, (d) 3-5 diCQA, (e) sucrose and (f) epicatechin in leaves submitted to photoperiod 12 h/12 h (orange line) and 8 h/16 h (red line). Dashed line represents a light period and the solid line represents the dark period. For all sugars and phenolics, values are expressed in mg 100 mg^−1^ dry matter, respectively. Error bars are means ± SEM of three experimental replicates.

Glucose and fructose contents were highly correlated for both photoperiods (*r* = 0.92, *P* < 0.01) and (*r* = 0.93, *P* < 0.01) for 8 h/16 h and 12 h/12 h photoperiods, respectively. Glucose and fructose patterns were similar during the day for the two photoperiods. During the night, the glucose and fructose production were higher under 12 h/12 h with peaks at ZT12 and ZT18 (Figure 7a and b). The leaf contents of glucose and fructose of plants grown under 8 h/16 h photoperiod appeared more stable during the whole diurnal cycle. Sucrose content was similar during the night for 12 h/12 h and 8 h/16 h photoperiods but at the beginning of the day (ZT3 and ZT6), sucrose content was higher at 8 h/16 h than under the 12 h/12 h photoperiod (Figure 7c).

The plants maintained in 12 h/12 h photoperiod presented a peak of accumulation of two classes of polyphenol precursors in the last 3 h of the light period (Figure S4 available as Supplementary Data at *Tree Physiology* Online; Figure 7). The content of two chlorogenic acids, 5-caffeoyl quinic acid (5-CQA) (Figure 7d) and 3–5 diCQA (Figure 7e) peaked at ZT9, and their levels were significantly higher (*P* < 0.05) than in leaves of plants grown under 8 h/16 h for the whole diurnal cycle. Flavanol (epicatechin) levels peaked at ZT12 (Figure 7f) and were significantly higher than those observed under 8 h/16 h at this time point, whilst for all other time points, levels were similar between both photoperiods.

### Genes clustering with the core clock genes *CaGI* and *CaLHY* and the photoreceptor *CaCRY1*

A cluster was considered as a group of oscillators that oscillate synchronously. Among all 100 clusters identified, three clusters contained the *CaLHY* and *CaGI* core clock genes or the *CaCRY1* photoreceptor. The photoperiod had an incidence on the composition of the clusters but the main associations remained present in both photoperiods, with 36–71% of the genes being common under both photoperiods (Table S7 available as Supplementary Data at *Tree Physiology* Online). For example, the correlation between *CaLHY* and chlorophyllide A oxygenase (*CaCAO*) remains constant (*r* = 0.87 for 12 h/12 h vs *r* = 0.89 for 8 h/16 h). As well, the correlation between *CaADO3* and *CaGI* was *r* = 0.86 at 12 h/12 h and 0.83 at 8 h/16 h, in agreement with the previous study showing that the FLAVIN-BINDING, KELCH REPEAT, F-BOX 1/ADAGIO3 (FKF1/ADO3) interacts with *GI* in a blue light-dependent manner (Imaizumi et al. 2005).

### Inferring gene regulatory networks

**Figure 8.**
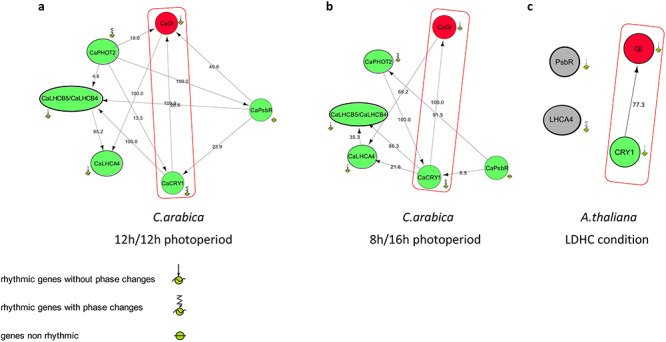
This figure shows the effect of Gigantea on the light phase of photosynthesis pathway. Networks have been inferred with a list of genes involved in the light phase of photosynthesis pathways and *CaGI* gene. (a, b) *C. arabica* networks have been inferred from rhythmic genes determined by JTK method. We used 12 h/12 h photoperiod data and 8 h/16 h photoperiod data. (c) Arabidopsis network has been inferred from Arabidopsis data of rhythmic genes determined with diurnal (Michael et al. 2008) in LDHC condition (light/dark cycles and hot (22 °C)/cold (12 °C) cycles). *CaPhot2*, *CaLHCB5* and *CaLHCB4* have not been included in the Arabidopsis network inference because they are not rhythmic in the ‘diurnal mocklerlab database.org’. The gene name written inside a red-filled circle indicates *GI* gene involved in circadian rhythm; gene names written inside a green-filled circle indicate genes involved in the light phase of photosynthesis. Ca = *C. arabica*. If two or more gene names are written on a node, it means that their expression profiles are correlated with Pearson correlation coefficient >85%. Bootstrap is specified in the middle of the arrow and lines. Gene names written inside a gray-filled circle indicate genes without interaction in the network. Rhythmic genes have been determined by JTK-DESeq2 method. The phase corresponds to the maximum value of the gene expression profile. If the phase occurs at the same time point in 12 h/12 h photoperiod and in 8 h/16 h photoperiod, rhythmic genes are called ‘rhythmic genes without phase change’. If the phase occurs at different time points in 12 h/12 h photoperiod and in 8 h/16 h photoperiod, rhythmic genes are called ‘rhythmic genes with phase change’.

Regulatory networks were inferred in order to investigate the effect of highly expressed core clock genes showing significant differences between both photoperiods, *CaLHY*, *CaELF4* and *CaGI*, on rhythmic genes encoding enzymes involved in four metabolic pathways: chlorophyll synthesis and degradation (seven genes), photosynthesis (Calvin–Benson cycle and light phase; 17 genes) and starch metabolism (12 genes) (Figures S1, S5 and S6 available as Supplementary Data at *Tree Physiology* Online). Five direct interactions involving those four metabolic pathways with a core clock gene were observed. They showed at least one existing interaction (bootstrap >79.4%) between the circadian gene and at least one gene of the metabolic pathway under consideration for both photoperiods (Table S5 available as Supplementary Data at *Tree Physiology* Online). A positive direct interaction between *CaCRY1* and *CaGI* was also inferred (Figure 8a and b). Previous studies in Arabidopsis already demonstrated that *CRY1* is involved in the regulation of *GI* mRNA cycling and post-transcriptional regulation of *GI* at dawn. Blue light acts through the blue light photoreceptors *CRY1* on *GI* expression in Arabidopsis (Paltiel et al. 2006). We also verified that this interaction existed from the data of Michael et al. (2008) (http://diurnal.mocklerlab.org/) for Arabidopsis (Figure 7c). Interestingly, in our case, the network also included several genes involved in the light phase of photosynthesis, suggesting that these two core clock genes do regulate the expression of a set of light phase photosynthetic genes.

## Discussion

Coffee is a tropical plant growing all over the year in almost constant 12 h/12 h photoperiods. In this study, we characterized coffee plants grown under their natural 12 h/12 h photoperiod, as well as in 8 h/16 h at the physiological, metabolic and transcriptomic levels. Next, we compared coffee responses with those of Arabidopsis, a temperate species that faces both photoperiods in its natural environment. We aimed to respond to one essential question: are photoperiod changes influencing on growth, metabolism and transcriptome? And if so, how does it differ from a temperate species such as Arabidopsis? Our results provide evidence that growth, secondary metabolism and global transcription differed between both photoperiods, but that for global transcription, these variations are not largely differ from those observed in Arabidopsis.

In total 1317 genes were differentially expressed between both photoperiods (*P*_adj_ < 0.05) at least one-time point (Figure 2), with for example 118 (or 148 genes with >twofold difference) at dawn, similarly to Arabidopsis which showed 40 genes with >twofold difference at dawn between the 8 h/16 h and 12 h/12 h photoperiods (Flis et al. 2016). A reduced duration of the day mostly altered the transcription before and after dawn (at ZT21-ZT0-ZT3) and at the end of the day or 4 h into darkness (ZT12) (Figure 2). On the other hand, the short-day had only a very limited effect during the rest of the day and in the middle of the night. If some genes showed large variations in expression between both photoperiods, the large majority did not show log-fold changes < 0.5 or > 2.0 (Table S2 available as Supplementary Data at *Tree Physiology* Online). The highest fold changes (>|2|) were mainly observed at ZT21 and the higher number of DEGs was observed at ZT3. When comparing dawn and dusk, we observed that photoperiod had a larger impact at dawn (Figure 2), similar to what had been previously observed in Arabidopsis (Flis et al. 2016). Many genes show rhythmic diurnal expression in Arabidopsis (e.g., 39% of the Arabidopsis transcriptome in an 8 h/16 h photoperiod; Michael et al. 2008). Interestingly, we found much less rhythmic genes in coffee, with 4129 genes (16%), of which 2859 were rhythmic in both photoperiods (Table S3 available as Supplementary Data at *Tree Physiology* Online). Thus, a large number of genes oscillating in Arabidopsis do not in coffee. Among the coffee rhythmic genes showing rhythmicity in both photoperiods (2859), thus being robustly identified, >89% were also rhythmic in Arabidopsis (Figure 5), demonstrating the strong similarity in the composition of the rhythmic diurnal gene set between both species. Interestingly, 85% of these genes were oscillating in constant light and cycling temperature condition in Arabidopsis, which suggests that diurnal regulation of gene expression in coffee is largely under the control of temperature. The identification of diurnally oscillating genes, which comprised several core clock genes (Figure 4) is an essential resource for future studies on coffee. When analyzing phase and amplitude changes of the rhythmic genes, it appeared that photoperiods influenced the phase and not the amplitude, with shorter phase in short photoperiod (Table S3 available as Supplementary Data at *Tree Physiology* Online; Figure 6a), in line with Arabidopsis, where short photoperiods lead to phase advance and anticipation of dawn (Flis et al. 2016). We then looked at *cis*-regulatory elements involved in clock regulation in order to see if these changes in phase could be regulated by the circadian clock. The EE element was clearly more altered in phase than the other elements (Figure 6b; Figure S2 available as Supplementary Data at *Tree Physiology* Online), which confirms that this element is essential to confer a time-of-day information (phase) (Michael and McClung 2002).

The short photoperiod modified the expression of important chloroplastic genes. Among the 130 genes present in the *C. arabica* chloroplast genome (Samson et al. 2007), we observed an overexpression of 37 genes at ZT21 just before dawn for the photoperiod 8 h/16 h (Table S6 available as Supplementary Data at *Tree Physiology* Online). Among those, Chlorophyll synthase (*CaCHLG*) and the chlorophyll b reductase (*CaNYC1*) have an essential role in the chlorophyll cycle and in the maintenance of the photosynthetic apparatus (Tanaka and Tanaka 2011; Figure 4). Interestingly, the chlorophyll content was significantly lower under short-days than under long days and we even observed a slight photobleaching on young leaves.

Because photoperiod influences gene expression and that such changes are partly driven by the circadian clock in Arabidopsis (Michael et al. 2008, Usadel et al. 2008), we looked at the network of coffee core clock genes. The main core clock genes and their connectivity were modified by the photoperiods (Figure 8). When we compared these networks to their counterparts in Arabidopsis (Flis et al. 2016, Michael et al. 2008), it appeared that coffee core clock genes (Figure 4a) showed similar cycling profiles to their Arabidopsis respective orthologues. Thus, coffee core clock networks seem to be adjusting to photoperiods. The photoperiod has a complex effect on core clock genes, with shifts in the phase of some (i.e., *CaLUX*) or the amplitude of other (*CaGI, CaLHY*) or both phase and amplitude (*CaPRR5, CaPRR7, CaELF4*). We observed significant variations of amplitudes for *CaPRR7*, *CaPRR5* and *CaGI* at ZT0. The regulation of plant clock function by metabolic status has been demonstrated for GI, which acts as part of the sucrose-signaling network in Arabidopsis (Dalchau et al. 2011). Endogenous oscillations in sugar levels provide also metabolic feedback to the circadian oscillator through the morning-expressed *PRR7* (Haydon et al. 2013). In line, we observed a higher content of glucose and fructose for coffee plants grown in 12 h/12 h during the night (Figure 7a and b). Consequently, the variations observed can come from both photosynthesis entrainment or cues transmitted by photoreceptors.

Short photoperiod influenced the expression of many genes (Figure 2) and in parallel, growth and leaves characteristics were altered (Figure 1). Among the dysregulated genes, we observed an enrichment in genes related to growth functions earlier in the day (ZT3) (Figure 3). We hypothesize that genes are expressed earlier under short photoperiods in order to maximize the use of the energy available directly from the light reactions of photosynthesis. However, we also observed some photobleaching in young leaves (Figure 1), which suggests that the plant in short photoperiod does not have the ability to handle higher light levels. In agreement, short-days led to a decrease in secondary metabolites involved in potent antioxidants. Under short photoperiods, the plants produced less polyphenol precursors (Figure S4 available as Supplementary Data at *Tree Physiology* Online; Figure 7). As well the lower expression of genes linked with flavonoid synthesis under short-days suggests that the photoperiod might modify the availability of these antioxidant compounds, which would have an impact on the capacity of coffee plants to cope with environmental stresses under high latitudes. Short-days might lead to a decrease in the accumulation of precursors necessary for the formation of complex polyphenols, lignin and condensed tannins. In leaves of plants maintained under an 8 h/16 h photoperiod, we observed an overexpression of genes involved in lignin synthesis, i.e., *CaPAL3*, *Ca4CL-L8* and *CaCAD7* at ZT3 and of *CaCCoAOMT1* at ZT21 (Figure S4 available as Supplementary Data at *Tree Physiology* Online). In contrast, the overexpression of *CaPAL3*, *CaF3H_1, CaDFR_2*, *CaANS*, *CaANR* and *CaLAR* argued in favor of a higher flavonoid synthesis under the natural 12 h/12 h photoperiod. Altogether, it suggests that a change in photoperiod has an impact on secondary metabolism, the 8 h/16 h photoperiod leading to overexpression of genes involved in lignin synthesis at the detriment of the synthesis of potent antioxidants (ROS scavengers) such as chlorogenic acids and flavanols.

## Conclusions

In this study, we investigated the incidence of short-days on the woody tropical perennial evergreen *C. arabica*, native form the southwest of Ethiopia (7° North) where the variations in the length of the day never exceed 30 min throughout the year. Currently, *C. arabica* is grown in areas where the differences in photoperiod length never exceed 2 h, suggesting this species might not be adapted to large changes in seasonal photoperiods. This study provides a comprehensive view of the incidence of a short photoperiod on the growth and transcriptome. Perhaps surprisingly, we found that coffee response to photoperiods had a lot of in common with Arabidopsis. However, variation in secondary metabolism, chlorophyll metabolism and light reactions of photosynthesis were identified. Such little difference could explain why this plant can be grown today in California (https://www.nbcsandiego.com/news/local/Could-Coffee-Be-The-Next-Cash-Crop-of-San-Diego--491399021.html). These results tend to prove that *C. arabica* cultivation can extend beyond the intertropical zone at latitudes up to the 35th parallel. In a previous study, we also showed that the allopolyploid nature of *C. arabica* also allowed it to withstand a relatively large range of growth temperatures, unlike its two parent species (Bertrand et al. 2015). However, studies remain to be done on coffee floral induction and bud break to ensure that short photoperiods do not affect productivity, these traits being species-and even often cultivars/ecotypes-dependent.

## Supplementary Material

Figure_S1_tpaa130

Figure_S2_tpaa130

Figure_S3_tpaa130

Figure_S4_tpaa130

Figure_S5_tpaa130

Figure_S6_tpaa130

Table_S1_tpaa130

Table_S2_tpaa130

Table_S3_tpaa130

Table_S4_tpaa130

Table_S5_tpaa130

Table_S6_tpaa130

Table_S7_tpaa130

Table_S8_tpaa130

Supplemental_figure_Legends_tpaa130

## Data Availability

All data generated or analyzed during this study are included in this published article and its supplementary information files.
