# Supplementary figures and images for: Photoperiod-dependent transcriptional modifications in key metabolic pathways in Coffea arabica
Source: Tree Physiol. 2020 Oct 20;41(2):302–16. doi: 10.1093/treephys/tpaa130 (PMC7874067; doi:10.1093/treephys/tpaa130)

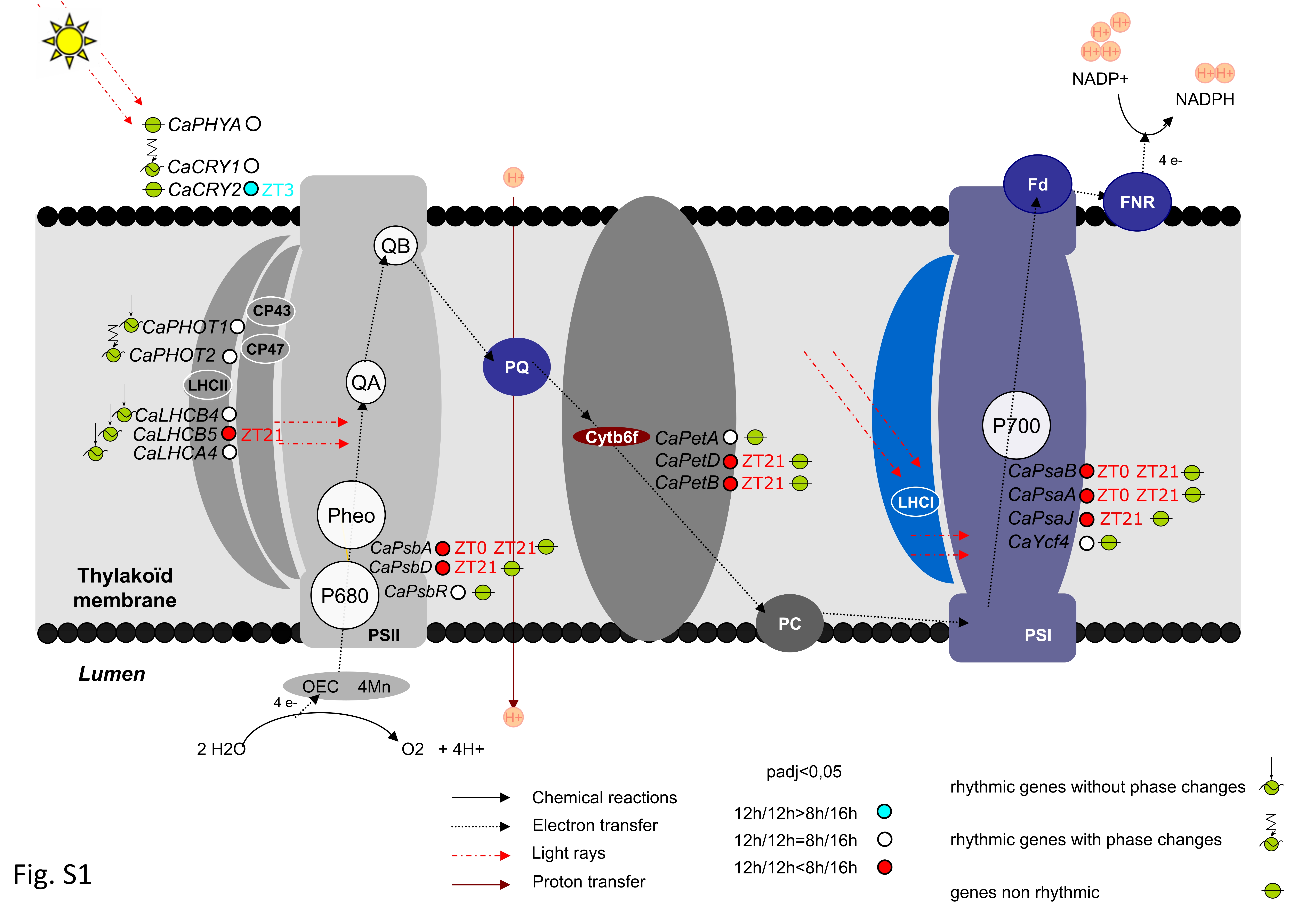

Supplement: Figure_S1_tpaa130 [file figure_s1_tpaa130.jpeg]

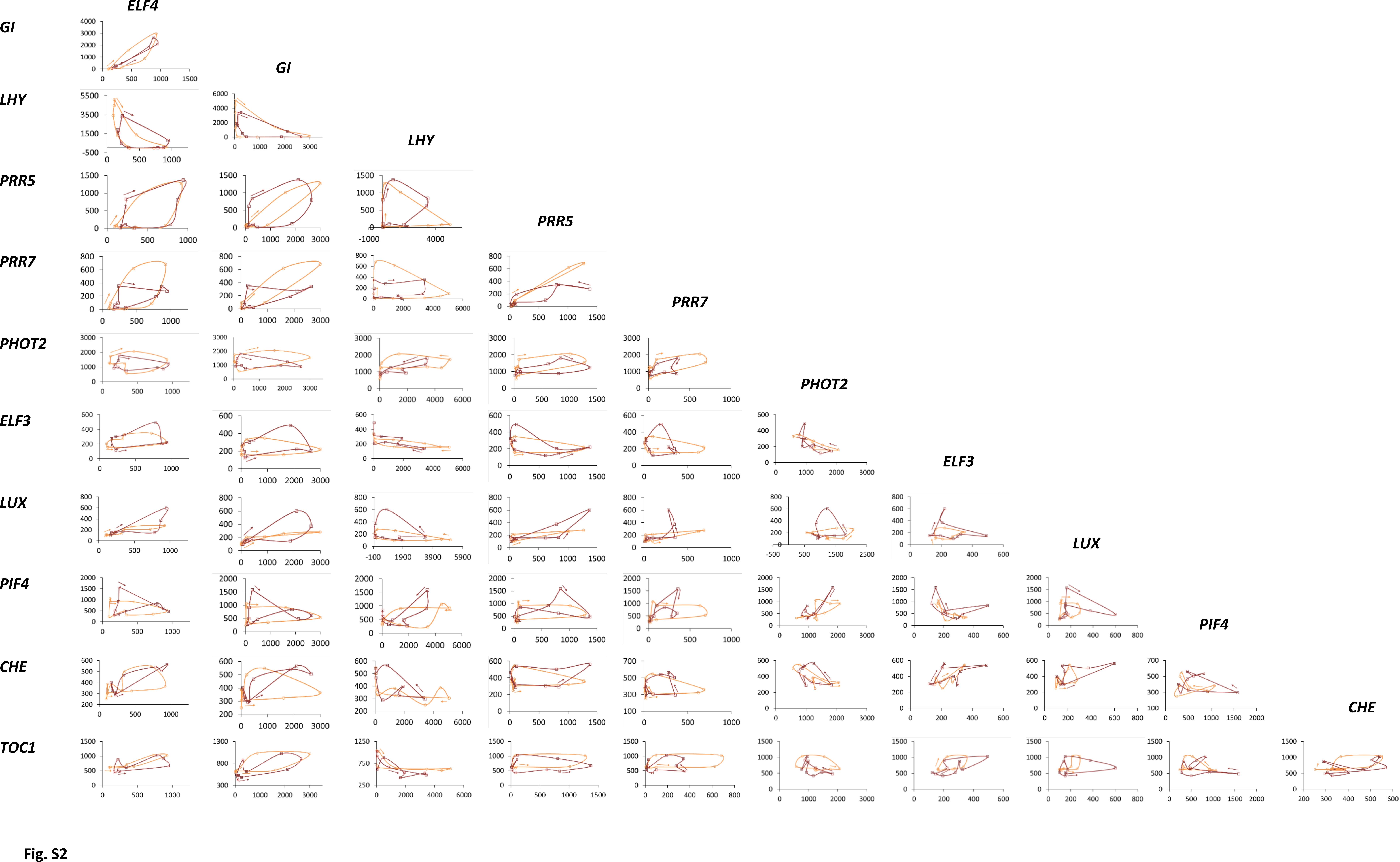

Supplement: Figure_S2_tpaa130 [file figure_s2_tpaa130.jpeg]

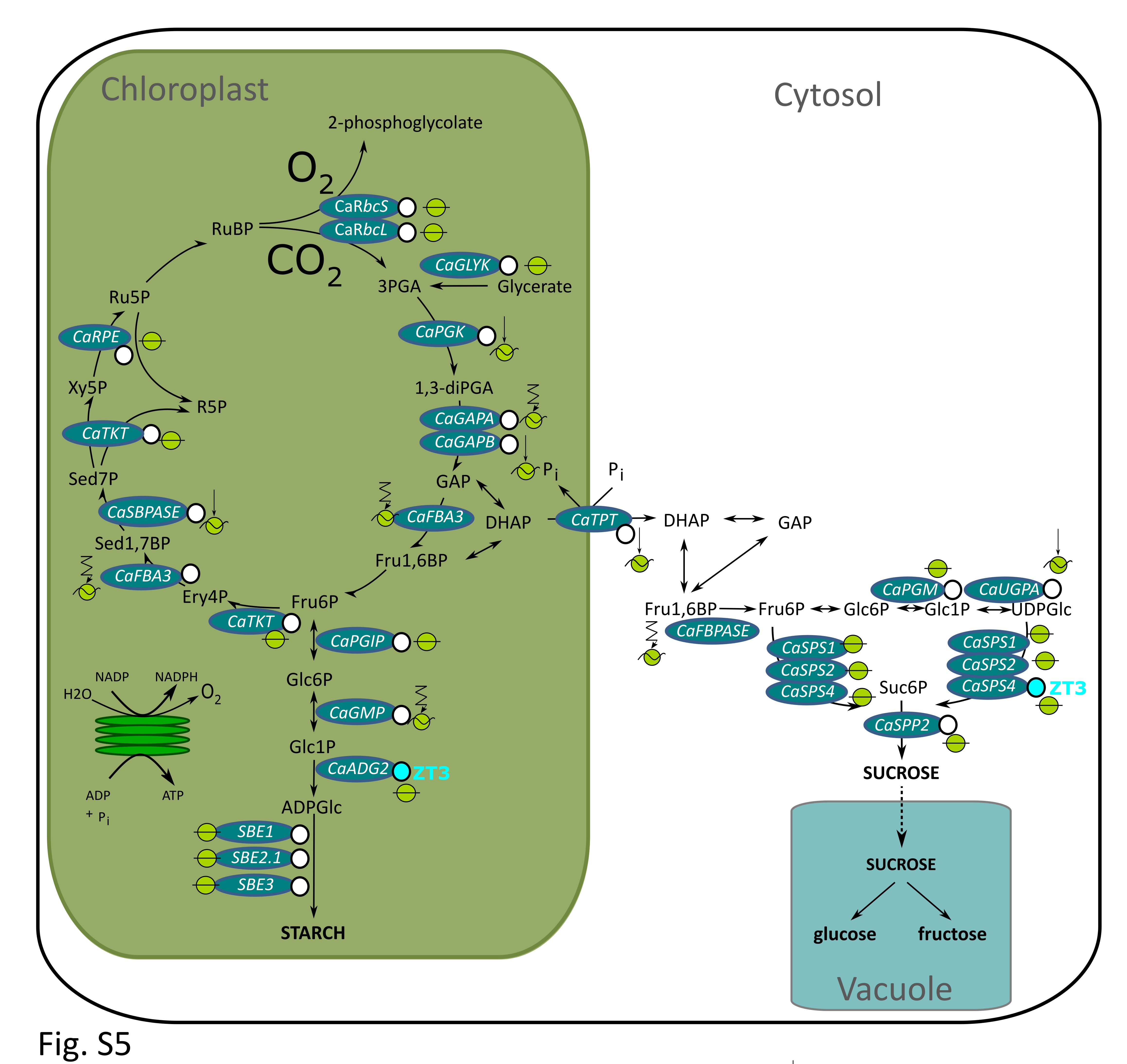

Supplement: Figure_S5_tpaa130 [file figure_s5_tpaa130.jpeg]

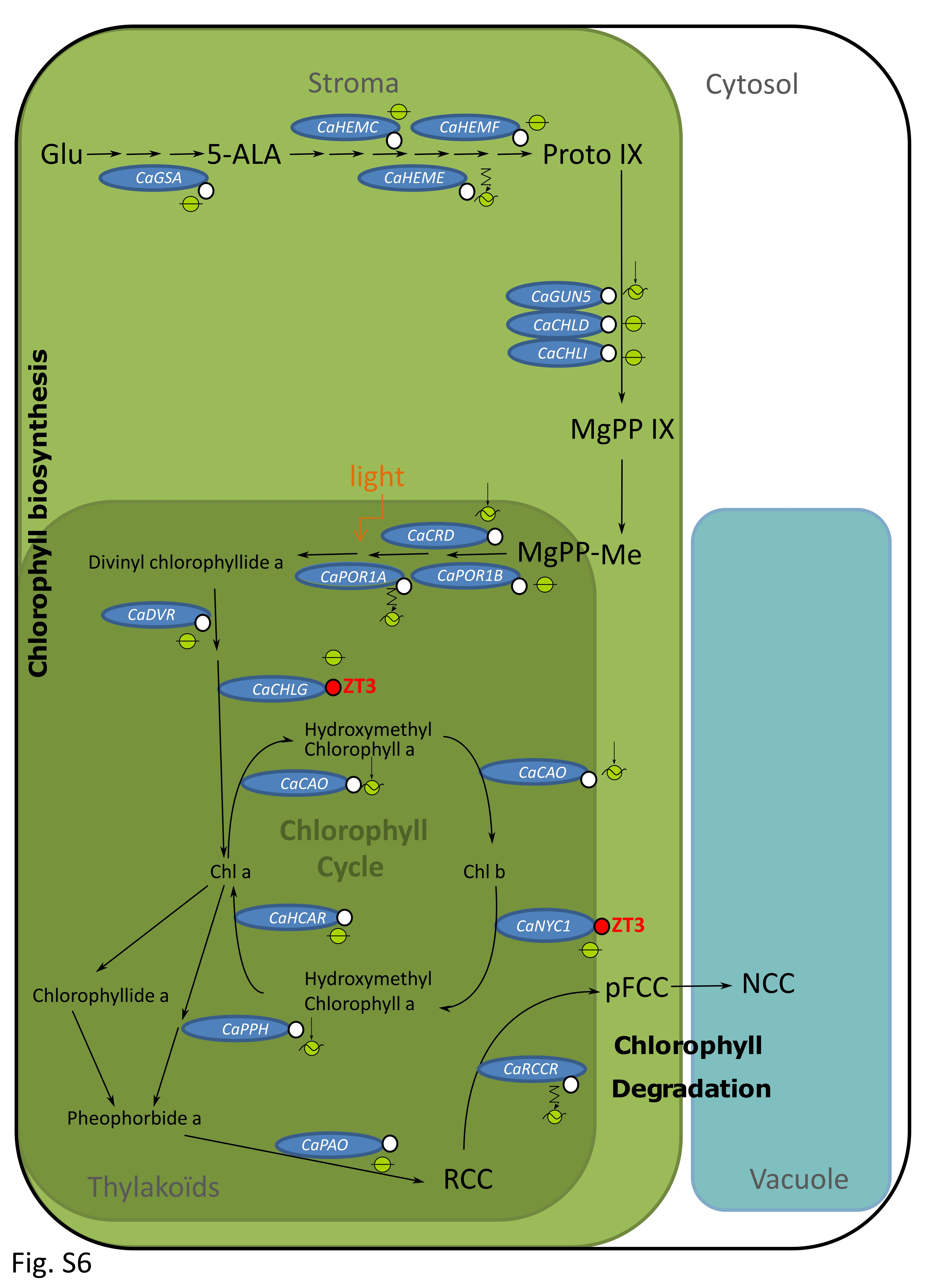

Supplement: Figure_S6_tpaa130 [file figure_s6_tpaa130.jpeg]
