# Supplementary material for: Photoperiod-dependent transcriptional modifications in key metabolic pathways in Coffea arabica
Source: Tree Physiol. 2020 Oct 20;41(2):302–16. doi: 10.1093/treephys/tpaa130 (PMC7874067; doi:10.1093/treephys/tpaa130)
Supplement: Supplemental_figure_Legends_tpaa130 [file supplemental_figure_legends_tpaa130.docx]

**Supplemental figure Legends**

**Fig. S1 Photoperiod dependent changes in the expression of genes involved in light signaling and the light phase of photosynthesis.** PSI(photosystem I), PSII(photosystem II), Cytb6f(Cytochrome bf complex) OEC: O2-evolving complex of photosystem II (PSII); 4Mn: 4 manganese atoms; O_2_: oxygen; H^+^: protons; P680: reaction center of PSII; Pheo: pheophytin; QA: PSII primary plastoquinone; QB: PSII secondary plastoquinone; PQ: mobile plastoquinone; PC : plastocyanin; P700: PSI chlorophyll reaction center; Fd: ferredoxin; FNR: Ferredoxin NADP Reductase; LHCII: light-harvesting complex II; CP47/CP43: antenna pigment-protein complex. Genes involved in this process are represented in italic and encode for: CaPHYA, phytochrome A(Cc02_g36930); CaCRY2, Cryptochrome 2(Cc03_g05480); CaCRY1, Cryptochrome 1(Cc10_g07160); CaPsbA, protein D1 (gene1 - chloroplast); CaPsbD, protein D2 (gene28 - chloroplast); CaPsbR, subunit R of PSII (Cc05_g15930); CaPetA, subunit f of cytb/f (gene57-chloroplast); CaPetD, subunit IV of cytb/f (gene77-chloroplast); CaPetB, subunit b6 of cb/f (gene76-chloroplast)); CaPsaA, P700 apoprotein A1 (gene36-chloroplast); CaPsaB, P700 apoprotein A2 (gene35-chloroplast); CaPsaJ, subunit IX of PSI (gene66-chloroplast); CaYcf4, PSI assembly protein Ycf4 (gene55-chloroplast); CaPHOT1, phototropin 1 (Cc01_g11930); CaPHOT2, phototropin 2 (Cc02_g31790); CaLHCB4, LHCII chlorophyll a/b binding protein 4(Cc06_g01460); CaLHCB5. LHCII chlorophyll a/b binding protein 5(Cc10_g16210); CaLHCA4, LHCI chlorophyll a/b binding protein 4(Cc04_g16410). The colored dots next to the genes indicate if there is a difference of expression between the two photoperiods (12h/12h vs 8h/16h); Blue dots are genes overexpressed under 12h/12h photoperiod; Red dots, genes overexpressed under 8h/16h and white dots are genes similarly expressed under both photoperiods. For the genes differentially expressed, the ZT when it occured is mentioned. p-value were adjusted (Benjamini-Hochberg) and significance considered at a threshold of 0.05.

**Fig. S2 Pairwise phase plots of the core clock genes.** The x- and y-axes represent the normalised transcript abundance. For each gene pair, the abundance of each transcript pair is plotted time point by time point. Sequential time points are connected by a line; arrow indicate the direction of the time vector,
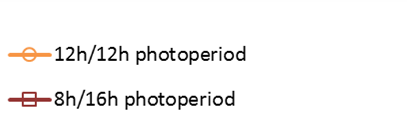
 indicates the 8h/16h photoperiod and
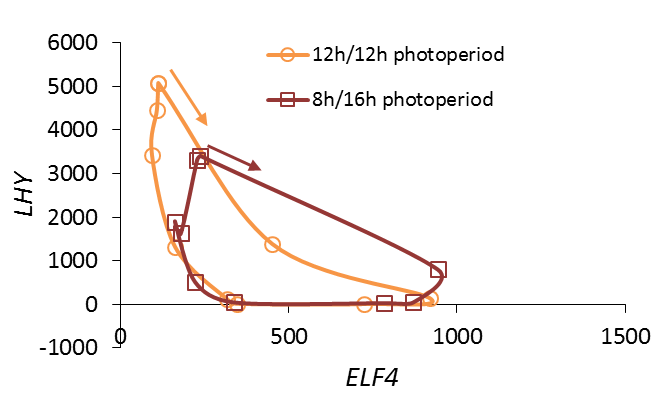
 indicates the 12h/12h photoperiod.

**Fig. S3 Time dependent enrichment in elements and functions for both photoperiods.** Radar chart which represent the number of rhythmic genes with cis-regulatory modules with a maximum value of the expression level through photoperiod 12h/12h (orange line) and 8h/16h (purple line). (**a**) EE= evening element, **(b**) and (**c**) CBS1 and CBS2= CIRCADIAN CLOCK ASSOCIATED 1-binding site (AA(A/C)AATCT), (**d**) PBX= protein box, (**e**) TBX= telobox, **(f**) ME= Morning element, (**g**) LBS= LUX binding site, (**h**) G-box= G-box, (**i**) SBX= sucrose box.

**Fig. S4 Heat maps of diurnal changes in transcripts abundance of genes involved in the phenolic compounds biosynthesis pathways in Coffea arabica.** Values are lfc of the comparison between 12h/12h photoperiod and 8h/16h photoperiod conditions. dark lines separate the homologues of two different genes. Ca = C. arabica. Cc = C. canephora. Framed values are the one with p-value adjusted by Benjamini-Hochberg < 0,05. ZT0 is equivalent to ZT24. rhythmic genes are highlighted.

**Fig. S5 Expression of genes involved in the second phase of photosynthesis.** NADPH  and ATP synthesized during the first photosynthesis phase are involved in CO2 fixation in the chloroplast through the Calvin-Benson cycle, thus enabling organic compounds production such as starch and sucrose respectively in the chloroplast and the cytosol. Genes involved in this pathway are represented in italic and they encode the following enzymes: ***CaRbcS*** = rubisco small Subunit(Cc00_g15710); ***CaRbcL*** = rubisco large Subunit(gene52(chloroplast gene)); ***CaPGK*** = phosphoglycerate kinase(Cc05_g16370); ***CaGAPB*** = NADP-Glyceraldehyde-3-Phosphate Dehydrogenase B(Cc10_g00410); ***CaGAPA*** = NADP-Glyceraldehyde-3-Phosphate Dehydrogenase A(Cc11_g00610); ***CaFBA3*** = plastidic aldolase 3 (Cc11_g09330); ***CaFBPase*** = cytosolic fructose-1,6-bisphosphatase(Cc10_g14740); ***CaTKT*** = transketolase(Cc02_g24060); ***CaSBPASE*** = sedoheptulose-1,7-bisphosphatase(Cc02_g06960); ***CaRPE*** = ribulose phosphate epimerase(Cc04_g15130); ***CaGLYK*** = glycerate kinase(Cc04_g03640); ***CaPGIP*** = plastidic phosphoglucose isomerase(Cc10_g15670); ***CaGMP*** = plastidic phosphoglucomutase(Cc02_g03610);***CaPGM*** = cytosolic phosphoglucomutase(Cc11_g08200); ***CaADG2*** = ADPglucose pyrophosphorylase(Cc03_g03330); ***CaSBE1*** = starch branching enzyme 1(Cc10_g00640); ***CaSBE2.1*** = starch branching enzyme 2.1(Cc06_g05400); ***CaSBE3*** = starch branching enzyme 3(Cc05_g14070); ***CaTPT*** = triose phosphate translocator(Cc02_g13860); ***CaUGPA*** = UDP-glucose pyrophosphorylase(Cc07_g12830); ***CaSPS1*** = sucrose phosphate synthase 1(Cc06_g07910); ***CaSPS4*** = sucrose phosphate synthase 4(Cc10_g16060); ***CaSPS2*** = sucrose phosphate synthase 2(Cc03_g08100); ***CaSPP2*** = sucrose-6'-phosphate phosphatase 2(Cc06_g06940). Ca = C. arabica. Cc = C. canephora. The colored dots near the genes indicate if there is a difference of expression between the two photoperiods (12h/12h vs 8h/16h); Blue dots correspond to overexpressed genes at the photoperiod 12h/12h and white dots represent genes without difference of expression between the two photoperiods. When the difference of expression occurs between the two photoperiods, the time expressed in hours after the sunrise (ZT) is mentioned next to the dots. Analyses were done with p-value adjusted by Benjamini-Hochberg and with a threshold of 0,03. ZT0 is equivalent to ZT24. (Figure adapted from Stitt et al., 2010).

**Fig. S6 Expression of genes involved in Chlorophyll biosynthesis and degradation for the two photoperiods in *Coffea arabica*.** The chlorophyll biosynthesis and degradation pathway occurs in Stroma, thylakoids and vacuole. All reaction steps are listed and genes involved in this process are represented in italic and they encode the following enzymes: ***CaGSA*** = Glutamate-1-semialdehyde 2,1-aminomutase(Cc10_g12640); ***CaHEME*** = Uroporphyrinogen decarboxylase(Cc05_g11120); ***CaHEMC*** = Porphobilinogen deaminase(Cc05_g16090); ***CaHEMF*** = Coproporphyrinogen-III oxidase(Cc09_g04450); ***CaGUN5*** = Magnesium-chelatase subunit H(Cc06_g17100***); CaCHLD*** = Magnesium-chelatase subunit chlD(Cc01_g06000); ***CaCHLI*** = Magnesium-chelatase subunit chll(Cc07_g18500); ***CaPOR1A*** = Protochlorophyllide reductase, chloroplastic(Cc05_g12370***); CaPOR1B*** = Protochlorophyllide reductase, chloroplastic(Cc05_g06850); ***CaCHLG*** = Chlorophyll synthase(Cc06_g01120); ***CaCAO*** = Chlorophyllide a oxygenase(Cc10_g11980); ***CaHCAR*** = 7-hydroxymethyl chlorophyll a reductase(Cc03_g07000); ***CaNYC1*** = chlorophyllide b reductase NYC1(Cc06_g09730); ***CaPPH*** = pheophytinase(Cc04_g14700); ***CaCRD*** = Magnesium-protoporphyrin IX monomethyl ester (oxidative) cyclase(Cc06_g22740); ***CaDVR*** = divinyl chlorophyllide a 8-vinyl-reductase(Cc03_g02320); ***CaPAO*** = Pheophorbide a oxygenase(Cc01_g10220); ***CaRCCR*** = Red chlorophyll catabolite reductase(Cc03_g11370). Ca = *C. arabica*. The colored dots near the genes indicate if there is a difference of expression between the two photoperiods (12h/12h *vs* 8h/16h); Red dots correspond to overexpressed genes at the photoperiod 8h/16h and white dots represent genes without difference of expression between the two photoperiods. When the difference of expression occurs between the two photoperiods, we mentioned the time expressed in hours after  dawn (ZT) next to the dots. Analyses were done with p-value adjusted by Benjamini-Hochberg and with a threshold of 0,02. ZT0 is equivalent to ZT24.
